# Synthesis, Purity Check, Hydrolysis and Removal of *o*-Chlorobenzyliden Malononitrile (CBM) by Biological Selective Media

**DOI:** 10.3390/toxics11080672

**Published:** 2023-08-04

**Authors:** Viorel Gheorghe, Catalina Gabriela Gheorghe, Daniela Roxana Popovici, Sonia Mihai, Catalina Calin, Elena Emilia Sarbu, Rami Doukeh, Nicoleta Grigoriu, Constantin Nicolae Toader, Cristiana Epure, Vasile Matei

**Affiliations:** 1 Department of Chemistry & Doctoral School, Faculty of Petroleum Refining and Petrochemistry, Petroleum-Gas University of Ploiesti, 39 Bvd. Bucuresti, 100520 Ploiesti, Romaniavasilematei@upg-ploiesti.ro (V.M.); 2 Research and Innovation Center for CBRN Defense and Ecology, Oltenitei 225, District 4, 041309 Bucharest, Romania

**Keywords:** *o*-chlorobenzylidene malononitrile, biological media, hydrolysis, biodegradation capacity, GC-MS, FTIR, CS

## Abstract

The removal yield of organic substances present in water depends on the environmental conditions, on the chemical composition of the water and on the chemical substance dissolved in the water, which constitutes the substrate of the metabolic activities of the microalgae that use these substances in the biochemical reactions of cellular enzyme complexes. *o*-Chlorobenzylidene malononitrile (CS, to use its military designation) was synthesized in-house, for research purposes, by a condensing reaction between *o*-chlorobenzaldehide and malononitrilein the presence of diethylamine. The detection, identification and confirmation of *o*-chlorobenzylidenemalononitrile (coded CBM in this experimental study) was performed using gas chromatography–mass spectrometry (GC-MS) and the purity of CBM was 99%. The biodegradation capacity in the samples that contained the biological suspension, after 24 h and 96 h of incubation, was determined via GC-MS analysis, and no evidence of the presence of CBM or some metabolites of CBM was detected. In the parallel samples, a hydrolysis process of CBM at room temperature, without biological treatment, revealed two main metabolites, malononitrile and *o*-chlorobenzaldehyde, respectively. This study is focused on evaluating the biodegradation capacity of *o*-chlorobenzylidene malononitrile in the presence of a biological material, culture of *Chlorella* sp., in comparison with a classical hydrolysis process. The tests performed indicate that the suspension of *Chlorella* sp. consumed the entire amount of CBM and metabolites from the analyzed samples. The tests prove that the biological material can be used to decontaminate the affected areas.

## 1. Introduction

As part of the riot control agent (RCA) category, halogenated tear agents cause acute physiological effects due their physical and chemical properties. The military taxonomy of class indices for chemical and biological warfare agents considers halogenated tear agents to be incapacitating, causing local pain and discomfort on inhalation, with associated reflexes. Among the adverse reactions generated by exposure to halogenated lacrimal agents is the “Kratschmer Reflex”, which causes tension of the aortic artery, referred to as apnea, due to the excitation of the sensory receptors of the nervous system [1].

Grenades containing *o*-chlorobenzylidene malononitrile have been used since the First World War [2,3,4]. Halogenated organic compounds are widely used for mass dispersion and for crowd control in incendiary devices. Due to their toxicity, there are concerns about the use of these substances on violent criminals but also on law enforcement agents, or other types personnel who are involved in such operations. These substances could also be used as a means of self-defense or for civil protection. The toxicity of the substances can affect anyone who is incidentally exposed, such as health care personnel, bystanders, noncriminals, or nonviolent offenders, leading to concerns about long-term or repeated exposure to halogenated toxicity [5].

*o*-Chlorobenzylidene malononitrile is a substance used in military operations that is used for crowd control. In explosives, in which it is found as a component of military projectiles, it is the chemical component of dispersive devices such as aerosolizers. The toxicological effects generated by this substance have serious consequences for the population and biofauna [5,6,7,8].

According to the Organization for the Prohibition of the Chemical Weapons (OPCW) document SAB-25/WP.1/27 March 2017, the Scientific Advisory Board (SAB) found that 17 substances meet the definition of an RCA as defined by Article II(7) of the Chemical Weapon Convention [9,10].

Halogenated organic chemicals, such as *o*-chloro benzylidene malononitrile, are included in the list of those 17 substances. *o*-chlorobenzylidenemalononitrile is also known by the synonyms *o*-chlorbenzalmalononitrile, K62 CS (pure), CS1 (95% CS, 5% silica aerogel), CS2 (CS and silica aerogel). CSX (1 g CS, 99 g tri-n-octylphosphite) is the most common RCA, known as “tear gas”. CS aerosol irritates the eyes, nose, and throat within 20–60 s, causing temporary disablement: tears, coughing, breathing difficulties, chest tightness, involuntary closing of the eyes, stinging of moist skin, mucous formation in the nose, and dizziness [11,12].

The medical problems generated by exposure to halogenated tear agents require a search for prophylactic antagonists to combat them because they are very persistent chemicals, remaining present in the environment for extended periods of time. The evaluation of the chemical risk induced by the persistence of chemical substances used in military operations requires the development of economically feasible methods of remediation of contaminated areas. The integration of effective depollution methods that decontaminate and, at the same time, reduce the risks of chemical pollution requires the use of biological methods. Natural environments, based on the bioavailability of microbiological suspensions capable of developing on selective media, should be involved in streamlining the performance of the process of eliminating toxic substances from the environment, and preventing the risk of new contamination due to military activities [6,7].

Dissemination of CS can be carried out on a targeted population in military operations involving explosive dispersion of powder or a solution containing CS, or via release in the form of smoke from a pyrotechnic mixture. The method of disseminating the toxin can generate a serious situation due to the severity of the injuries to the eyes or the respiratory system of the affected subjects [13,14,15,16,17,18,19]. CS tends to agglomerate when used in powder form, which is why hydrophobic formulations of CS have been developed that contain siliconized powder including a hydrophobic silicon aerogel. This allows the active substance to have a long-lasting effect, persisting for up to several weeks [20].

In the presence of pollutants from the irritating substances category, it is necessary to adapt the microbial cultures and test the biodegradation capacity of these toxins, taking into account their speed and detoxification capacity, as well as the cellular toxicity exerted by the harmful substance in a biological environment [21,22].

Biological suspension can influence the biodegradation of chemical compounds through its genetic ability to metabolize organic substances in close correlation with the rate of assimilation of the toxicant. To accelerate these processes, certain optimal conditions for cell development are needed (pH, temperature, nutrient substrate, agitation, incubation, etc.) [23,24,25,26].

The toxic substances present in water influence the enzymatic system of algae. A very important aspect in the biodegradation of toxic compounds is the chemical nature of the substance, which determines the persistence of the compound in the reaction medium. The contact time of microorganisms from a biocenosis with a toxic substance is important; the retention time and biodegradation capacity lead to an increased effect of the metabolism of toxic substances, which increases the reaction speed in the tested environment [23].

According to the MICHAELIS–MENTEN relationship in enzyme kinetics, it was established that the speed of biochemical processes in the cell increases with the increase in the concentration of the substrate until a lag phase is reached, in which the cell growth no longer changes in relation to the concentration of the substrate. Following this process, percentages of the undegraded substance remain in the reaction medium, and metabolites resulting from biochemical processes also accumulate [27].

The biochemical processes that take place in the cells of algae are mechanisms that can vary at different speeds because chemical substances can be attacked sequentially, generating a sequential increase due to some metabolism products. The metabolic reactions involved in the cells of microorganisms are catabolic, meaning that the oxidation of substances takes place with the purpose of obtaining energy and anabolism or assimilation, in which microorganisms use the energy resulting from degradation reactions [28].

The cellular biochemical mechanism of Chlorella algae is based on their ability to use sunlight as an energy source for CO_2_ fixation, the production of photosynthesis, a process through which the biomass provides the oxygen necessary for the biodegradation of organic substances. This helps to increase the cell mass by using CO_2_ as a carbon source, biomass development being favored by environments with alkaline pH after the reaction 2HCO^3−^ → CO_2_ + CO_3_^2−^ + H_2_O [29,30,31].

In our previous tests, we performed a series of ecotoxicity tests on the CBM substance using different microbial cultures. CS toxicity was highlighted by testing CBM solutions of different concentrations brought into contact with suspensions of microalgae *Chlorella* sp., yeast *Saccharomyces* sp., *microorganisms Lactobacillus* sp. and the ciliate *Paramecium* sp. It was observed that the most effective biological strain for CBM degradation it was *Chlorella* sp. because it had the best cell development and regeneration under increasing concentration conditions, from 10 µg/mL to 150 µg/mL [32].

After the tests, it was observed that the cellular inhibition of *Chlorella* sp. was due to the oxidative stress to which they were subjected, which generated a change in the growth of biomass compared to the control sample that was treated under identical conditions but without toxic. In other experiments, we tested the toxic effects of CBM through studies of ecotoxicity on *Barbus Capoeta Tetrazone* fish, which highlighted the fact that the toxicity limits were established by LC_50_ estimated at 24 h 2.9 mg/L CBM and LC_50_ estimated at 72 h 1.2 mg/L CBM [28].

## 2. Experimental Design, Materials and Methods

### 2.1. Aspects on the Synthesis Reaction of the o-Chlorobenzylidene Malononitrile (CBM)

**Figure 1:**
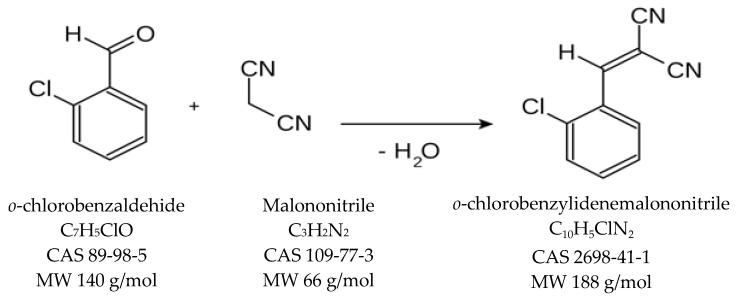
The synthesis reaction of the *o*-chlorobenzylidene malononitrile.

The following reagents were used in the synthesis reaction of *o*-chlorobenzylidene malononitrile: *o*-chlorobenzaldehide (C_7_H_5_ClO, CAS Registry Number 89-98-5, MW 140 g/mol, Thermo Scientific, 97% purity), malononitrile (C_3_H_2_N_2_, CAS Registry Number 109-77-3, MW 66 g/mol, Sigma-Aldrich, 99% purity) and diethylamine (C_4_H_11_N, MW 73 g/mol, CAS Registry Number 109-89-7, Sigma-Aldrich, 99.5% purity) as a catalyst. *o*-Chlorobenzylidene malononitrile was synthesized in-house via a condensing reaction between *o*-chlorobenzaldehide and malononitrile (Figure 1), in the presence of diethylamine [33,34,35,36].

**Figure 2:**
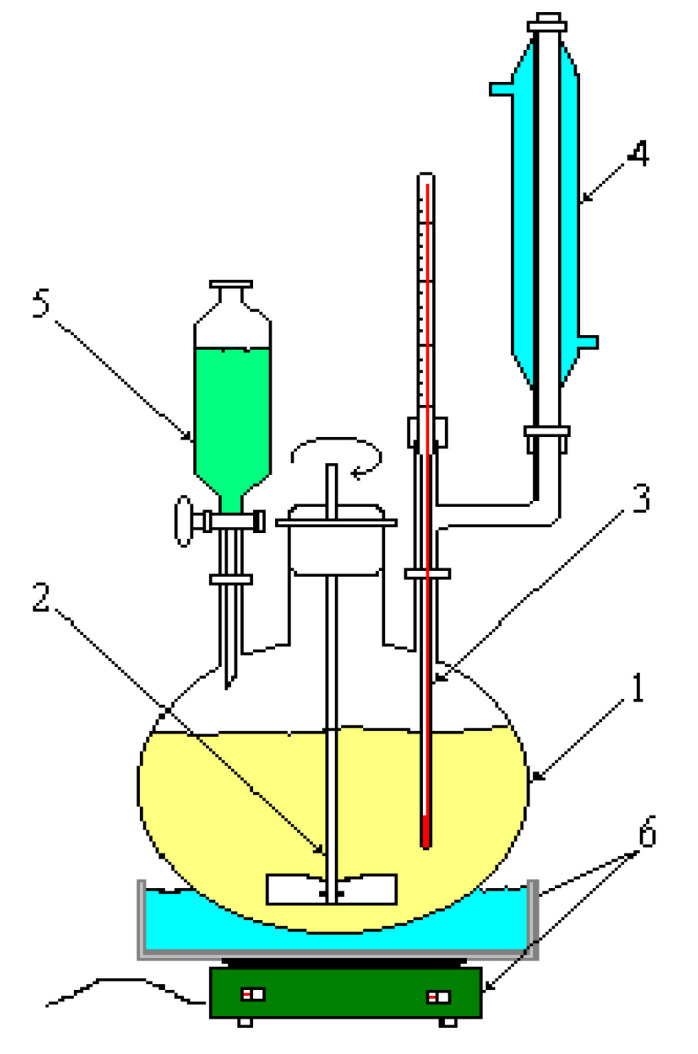
The working installation for the synthesis of CBM (1—round-bottom balloon, 2—mechanical stirrer, 3—thermometer, 4—refrigerant, 5—drip funnel, 6—heating plate).

The two -CN groups in the malonic nitrile molecule are strongly electron-attracting and activate the CH_2_ group to which they are linked, and because of this, malononitrile plays the role of a methylene component in condensation reactions with aldehydes or ketones. The reaction takes place in the presence of catalysts such as secondary or tertiary amines (piperidine, diethylamine, etc.). In the synthesis reaction of CBM, we used diethylamine. The working installation for the synthesis of CBM is presented in Figure 2. The purity of CBM was checked via gas GC-MS in methylene chloride (DCM, Supra Solv, purity of 99.8%) as an organic solvent [37].

### 2.2. Preparation of Biological Medium

An analytical balance OHAUS model AX224M was used to prepare the CBM solutions and the culture medium for the development of the algal suspension. The monitoring of cell viability during the experiment was carried out using a CELESTRON Microscope, model 4434. The biological material used in the experiments was *Chlorella* sp., which was obtained from the Culture Collection of Algae of Petroleum Gas, University of Ploiesti, and it was adopted as model organism for experiments.

Microalgae *Chlorella* sp. was cultivated in Erlenmeyer flasks containing medium (previously sterilized by autoclaving for 15 min at 121 °C), (250 cm^3^ of distilled water, MgSO_4_·7H_2_O (0.3 g), KNO_3_ (0.4 g), CaCl_2_ (0.4 g), NaH_2_PO_4_·2H_2_O (0.3 g), FeSO_4_·7H_2_O (0.02 g), NaNO_3_ (0.3 g), NH_4_Cl (0.2 g); pH was adjusted at 6.5).

Erlenmeyer flasks were placed in a laboratory shaker (ORBITAL Multi-Shaker) at 100 rpm for optimal aeration at 35 °C ± 1 °C, incubation under continuous white light (intensity was in the range 60–120 µE∙m^−2^∙s^−1^) [32].

### 2.3. Calibration Curve of o-Chlorobenzylidene Malononitrile and o-Chloro Benzaldehide

The dilution series of CBM in DCM (µg/mL), sample code: S01CBM 2000 (µg/mL), S02CBM 300 (µg/mL), S03CBM 200 (µg/mL), S04CBM 150 (µg/mL), S05 CBM 100 (µg/mL), S06CBM 50 (µg/mL), S07CBM 20 (µg/mL), S08 CBM 10 (µg/mL), S09CBM 4 (µg/mL), S10CBM 2 (µg/mL).

The dilution series of *o*-chlorobenzaldehide in DCM (µg/mL), sample code: S01CB 2700 (µg/mL), S02CB 189 (µg/mL), S03CB 135 (µg/mL), S04CB 81 (µg/mL), S05CB 54 (µg/mL), S06 CB 27 (µg/mL), S07CB 13b (µg/mL), S08 CB 5.4 (µg/mL), S09CB 2.7 (µg/mL).

#### Method Quantification

A Thermo Electron chromatograph GC Trace 1310 equipped with a mass detector TSQ 9000 was used for the study. The quantification of the chemicals of interest, CBMand *o*-chlorobenzaldehide, was based on a 10-point calibration curve (for CBM) and a 10-point calibration curve (for *o*-chlorobenzaldehide) obtained by plotting the area ratio of the target compounds and concentration of the calibration standards.

The range of the calibration curve used for CBM identification was located in the domain of 2000–2 µg/mL and that used for *o*-chlorobenzaldehide identification was located in the domain of 189–2.7 µg/mL.

Excellent linearity of response was observed over the concentration range from 2 to 300 µg/mL for CBM-C3 (calibration curve equation: y = 3e + 07x + 2e + 08, R^2^ = 0.9998) and 2.7 to 189 µg/mL for *o*-chlorobenzaldehide (calibration curve equation: y = 3e + 07x + 5e + 08, R^2^ = 0.9994).

The method for quantitative determination of CBM and *o*-chlorobenzaldehide was pre-validated within the laboratory procedure “Quantitative determination of irritant-lachrymatory agents from various types of samples”, in accordance with ISO/IEC 17025 and Recommended Operating Procedures for Analysis in the Verification of Chemical Disarmament. The Ministry of Foreign Affairs of Finland, University of Helsinki (2017) [38].

### 2.4. Preparation of the Bioreactors with Biological Samples Contaminated with CBM

To evaluate the toxicity of CBM on the culture of *Chlorella* sp., solutions of different concentrations of CBM were prepared via dissolution in water. CBM concentrations were prepared via ultrasonic dispersion using an Ultrasonic SONICA S3—Soltec model.

In the tests, 2 replicate series were used (series A and series B), each series contains 7 test tubes (bioreactors) coded PA1-PA7, PA10, respectively PB1-PB7, PB10. In all test tubes were added 2 mL (10^4^ ufc/mL) from the algal suspension *Chlorella* sp. in the phase of exponential growth. PA10 and PB10 were diluted to 10 mL with distilled water and marked as blank. The rest of the test tubes were loaded up to 10 mL with the established concentrations of CBM, obtaining the following solutions in the bioreactors: PA1 (20 µg/mL), PA2 (40 µg/mL), PA3 (60 µg/mL), PA4 (80 µg/mL), PA5 (100 µg/mL), PA6 (120 µg/mL), PA7 (140 µg/mL), respectively PB1 (20 µg/mL), PB2 (40 µg/mL), PB3 (60 µg/mL), PB4 (80 µg/mL), PB5 (100 µg/mL), PB6 (120 µg/mL), PB7 (140 µg/mL). The tubes were incubated for 96 h, followed by mechanical stirring in an ORBITAL SHAKER (100 rpm) and were kept at a temperature of 35 °C under white light (intensity was in the range 60–120 µE∙m^−2^∙s^−1^).

### 2.5. FTIR Analysis of Biological Samples

After 24h from the incubation of the biological samples, from series A (PA1-PA7) and PA10 blank, FTIR characterizations were performed using the TRACER IR spectrophotometer (Shimadzu IR TRACER-100, Kyoto, Japan) in the region of 4000–400 cm^−1^. Each peak was assigned a functional group. FTIR spectra have distinct bands and were assigned a range of vibrationally active chemical groups [39,40,41].

Vibrational spectroscopy has been used to study the chemical composition of biological suspension for samples coded PA1-PA7, PA10 blank. After the FTIR analysis, the samples were incubated for up to 96 h.

### 2.6. Chemical Characterization and Confirmation of the Synthesized Compound CBM by GC-MS

The detection, identification and confirmation of the interest chemicals, CBM and *o*-chloro benzaldehide, was performed via GC-MS, in accordance with the laboratory procedure “Separation and identification of chemical compounds with military relevance”, accredited by Romanian Accreditation Association (RENAR), in accordance with ISO/IEC 17025.

A Thermo Electron chromatograph GC Trace 1310 equipped with a mass detector TSQ 9000 was used for the study. The GC was fitted with a 15 m × 0.25 mm i.d. TR-5MS bonded phase column (5% Phenyl Methyl Siloxane), 0.25 µm film thickness (Thermo Electron Corporation, Waltham, MA, USA). Helium (99.9999 purity) was used as the carrier gas with a flow rate of 1 mL/min. (36.2 cm/s) constant pressure. The oven temperature was held initially at 40 °C for 1 min, programmed from 40 °C to 250 °C at 10 °C min^−1^, and held at 250 °C for 1 min. Splitless injections of 1 µL volume were performed using a Thermo Electron Corporation AI800 autosampler [42,43,44].

The compound was identified based on the reference chemical from the NIST Database [34]. The spectrometric technique used was mass spectrometry, in electron impact ionization (EI) mode, for a mass range between 40 and 650 amu. The electron ionization MS operating conditions were as follows: ion source pressure approximately 1.5 × 10^−5^ torr; source temperature, 250 °C; electron energy, 70 eV; and electron multiplier voltage +400 V relative to the autotune setting.

Biological samples were prepared via extraction of 10 mL of the incubated water sample with 3 mL of DCM via manual agitation for 10 min. The organic extract was dried on sodium sulphate anhydrous for 1 h, filtered through a HPLC filter unit (0.45 µm, Millipore Millex-HV), transferred to salinized glass autosampler vials and analyzed via GC-MS. A water sample which was not spiked with CBM, solely containing the biological suspension, was used as blank (sample code P10blank).

From series A [(PA1–PA7) and PA10 blank] after 96 h of incubation and from series B [(PB1–PB7) and PB10 blank] after 24 h of incubation, the metabolites resulting from the solubilization of the substance were analyzed via GC-MS and the degree of hydrolysis of CBM in aqueous solution was determined.

### 2.7. Hidrolysis Analysis

In an aqueous, neutral environment, CBM is relatively stable during hydrolysis. Through hydrolysis, the double ethylenic bond is broken with the formation of *o*-chloro benzaldehyde and malonic nitrile, the reverse reaction to obtain CBM.

The study conducted to evaluate the hydrolysis reaction of CBM in an aqueous environment was carried out by dissolving a known amount of CBM in a known volume of tap water; the samples were processed in the time interval 30 min–24 h, reported to a reference t_0_ and analyzed using the GC-MS chromatographic system.

## 3. Results

Chemical substances are degraded through different metabolic pathways, through the oxidation of carbon and hydrogen from organic substances, through the oxidation of nitrogen from nitrites or from chemical substances that contain nitrogen in the molecule, hydrolysis or the removal of water at C=C atoms via addition to the double bond, splitting and forming C-C bonds by decarboxylating or carboxylating ketones and adding or removing the N atom in the form of NH_3_. In addition to the metabolic reactions of the cells, there are also reactions by which the toxic organic substances are inactivated and eliminated from the reaction medium through methylation, acetylation [27].

### 3.1. Detection, Identification and Confirmation of CBM by GC-MS

The CBM of 99% purity was synthesized in the laboratory according to the description in Section 2.1. Following the GC-MS analysis, three other substances were identified in a percentage of 1%, according to Table 1, coded as follows: *o*-chlorobenzaldehydes (C1), *o*-chlorobenzylmalononitrile (C2), *o*-chlorobenzylidene malononitrile (CBM-C3) and 2-(3-chlorobenzylidene) malononitrile (C4) [44].

**Figure 3:**
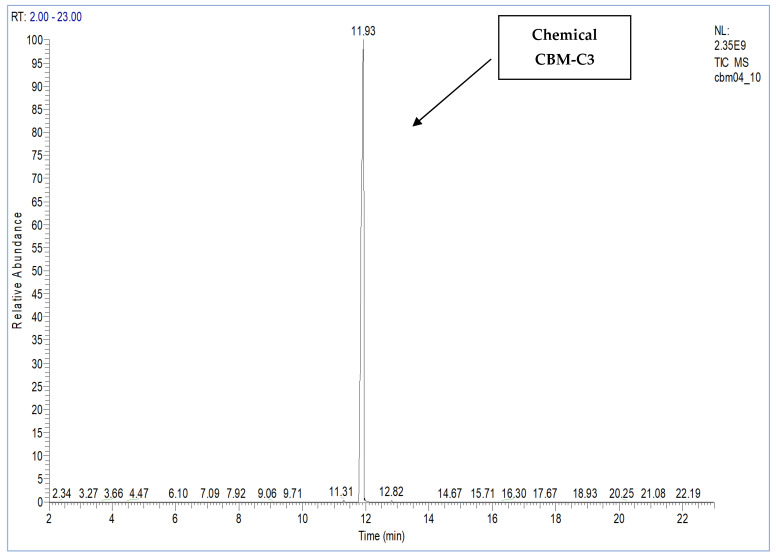
The total ion chromatogram for the sample containing 2-chloro benzalmalononitrile (CBM-C3) (retention time of 11.93 min), sample code CBM04.

**Figure 4:**
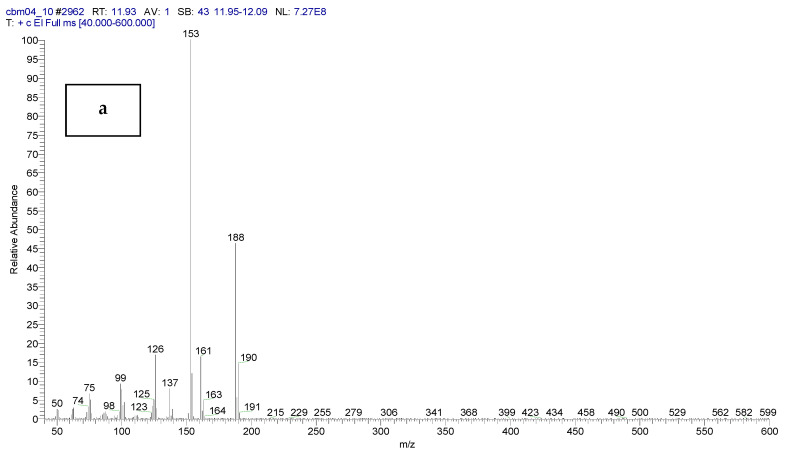

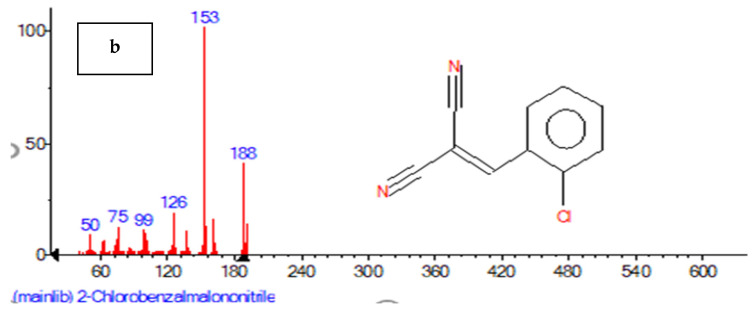
EI mass spectra of CBM-C3—own synthesis—(**a**), compared with the reference chemical from the NIST database (**b**).

The following figures show the total ion chromatogram (Figure 3), the EI mass spectra (Figure 4) of the synthesized chemical CBM-C3 (retention time of 11.93 min.) and the reference chemical from the NIST Database. The concentration used for CBM-C3 identification was 150 µg/mL (sample code CBM04) [34].

**Figure 5:**
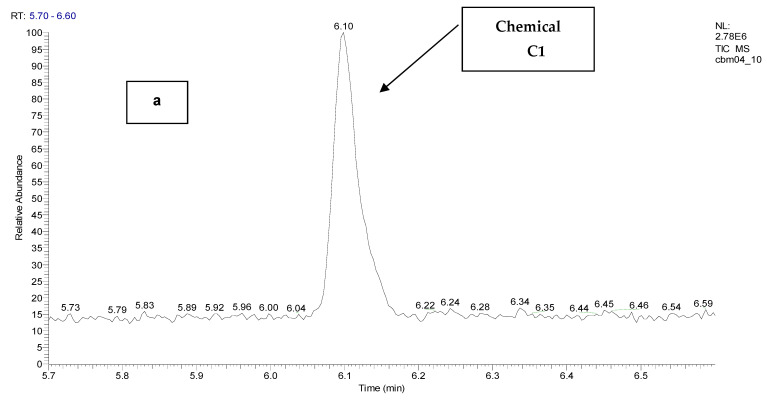

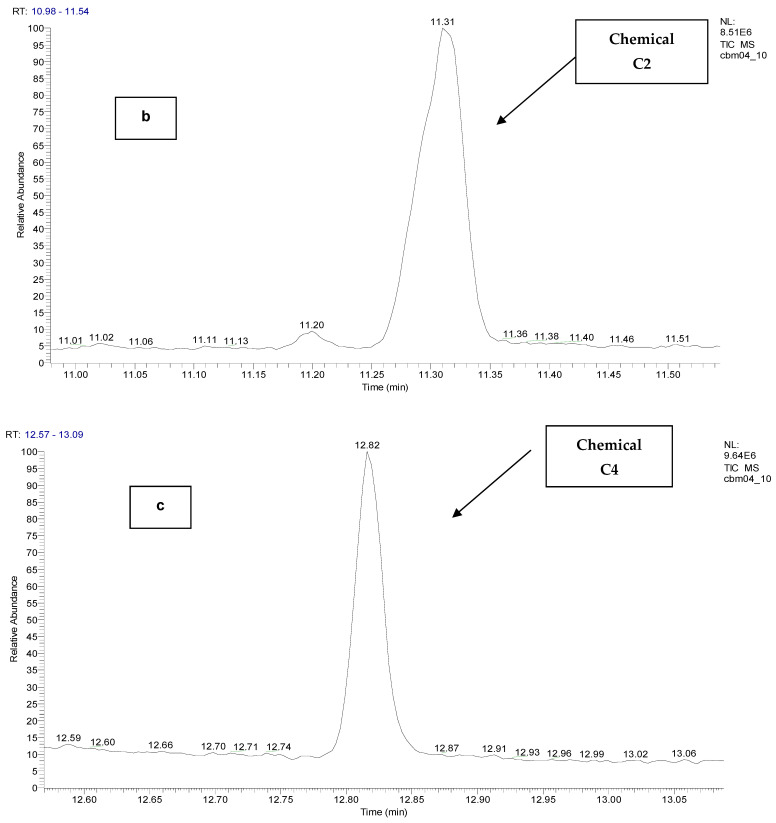
EI chromatograms supporting identification of: (**a**) *o*-chlorobenzaldehide (C1) retention time of 6.10 min; (**b**) *o*-chlorobenzylmalononitrile (C2) retention time of 11.31 min; (**c**) 2-(3-chloro benzylidene) malononitrile (C4), retention time of 12.82 min.

**Figure 6:**
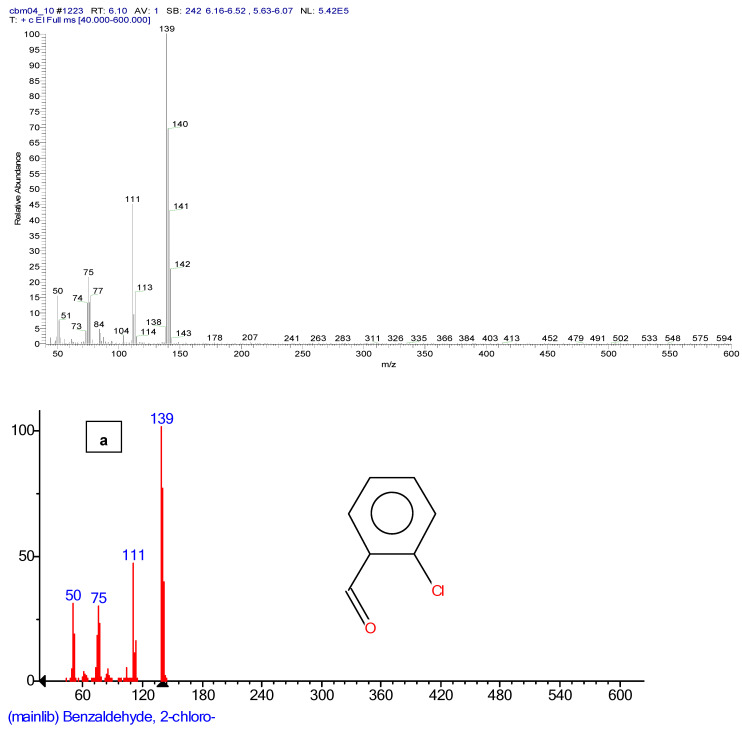

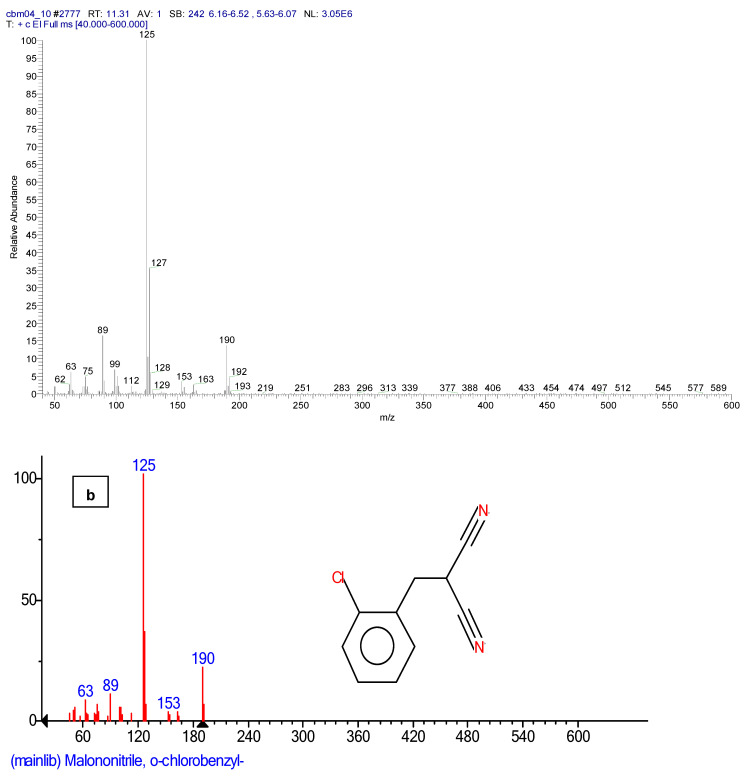

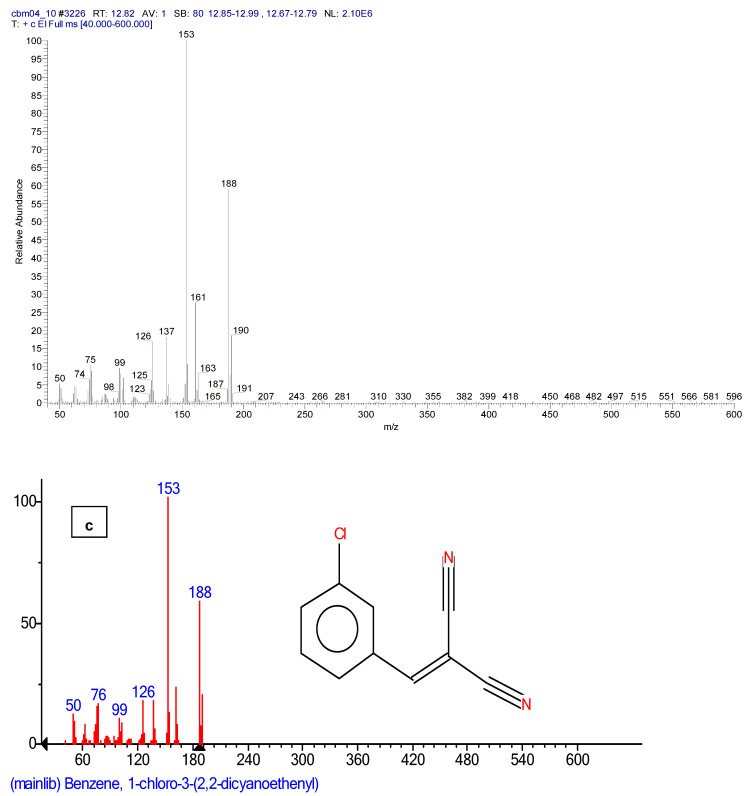
EI mass spectra of: (**a**) *o*-chlorobenzaldehide (C1); (**b**) *o*-chlorobenzylmalononitrile (C2); (**c**) 2-(3-chloro benzylidene) malononitrile (C4), compared with the reference chemical from the NIST database.

EI chromatogram supporting identification of *o*-chlorobenzaldehide (C1), retention time of 6.10 min, *o*-chlorobenzylmalononitrile (C2) retention time of 11.31 min, and 2-(3-chloro benzylidene) malononitrile (C4), retention time of 12.82 min, the other component of the reference chemical, 2-chlorobenzalmalononitrile (CBM-C3), are presented in Figure 5, and the mass spectra of the chemicals, in comparison with the references from the NIST database are presented in Figure 6.

### 3.2. Extraction of the Spiked Samples with CBM and Biological Suspension and Analysis by GC-MS and FT-IR

The codes of the GC-MS investigated samples corresponding to the biological suspensions marked with CBM are as follows: PA196EI to PA796EI corresponds to PA1-PA7 samples and PB124EI to PB724EI corresponds to PB1-PB7 samples.

**Figure 7:**
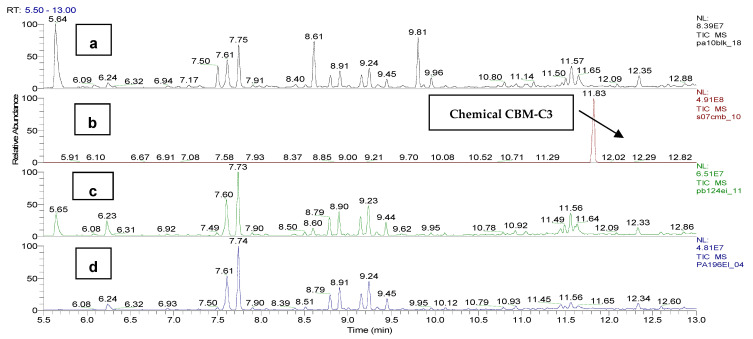
EI total ion chromatogram of: (**a**) blank sample, coded PA10BLK; (**b**) CMB solution of 20 µg/mL, sample code S07CBM; (**c**) solution of 24 h incubation, sample code PB124EI; (**d**) solution of 96 h incubation, sample code PA196EI. CBM-C3 has the retention time of 11.83 min.

In Figure 7, we compare the blank sample, coded PA10BLK, which means the biological suspension without CBM, the CBM dilution of 20 µg/mL, sample code S07CBM, and the sample with the microalgae and CBM at 20 µg/mL, with 24 h incubation, coded PB124EI and with 96 h incubation, coded PA196EI.

The graph shows the absence of the peak of the chemical *o*-chlorobenzaldehide (C1) with a retention time of 6.10 min in the samples PB124EI and PA196EI, no peak of the chemical CBM-C3 (retention time of 11.83 min) in the samples PB124EI and a trace level in the sample PA196EI, with no peak signal for the substances *o*-chloro benzylmalononitrile (C2) and 2-(3-chloro benzylidene)malononitrile (C4).

**Figure 8:**
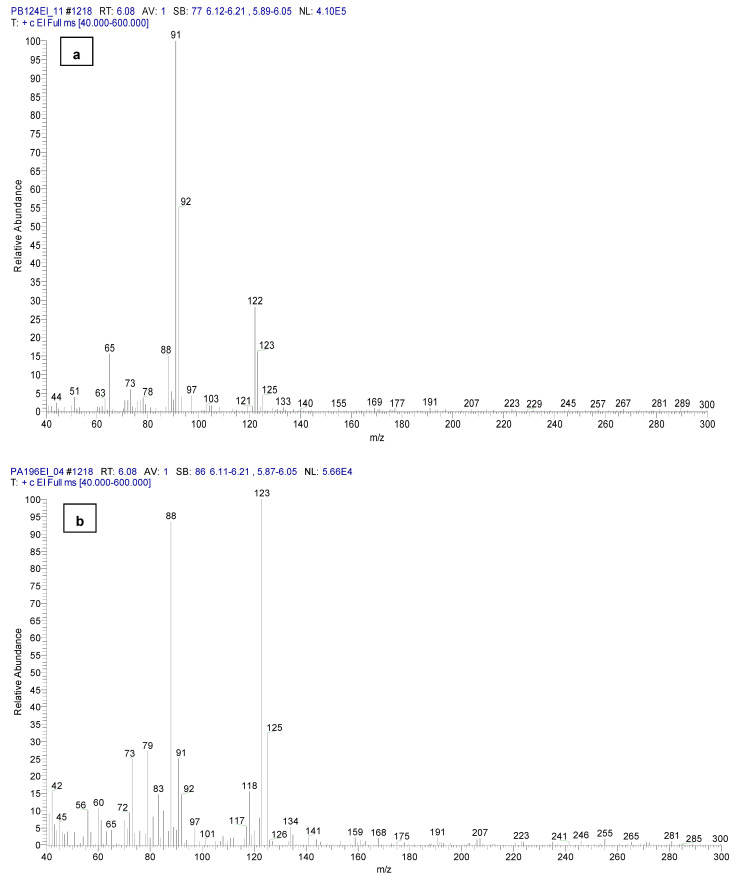
Mass spectra from the samples: (**a**) PB124EI and (**b**) PA196EI (the absence of the *o*-chlorobenzaldehide (C1) retention time of 6.08 min).

In the sample reference of CBM, sample code S07CBM, at the retention time of 6.10 min, a minor chromatographic peak was identified that supports the identification of *o*-chlorobenzaldehide (C1) based on the synthesis reaction. The absence of this chemical from the sample codes PB124EI and PA196EI is proved by the mass spectra of the chromatographic peaks at 6.08 min (Figure 8).

**Figure 9:**
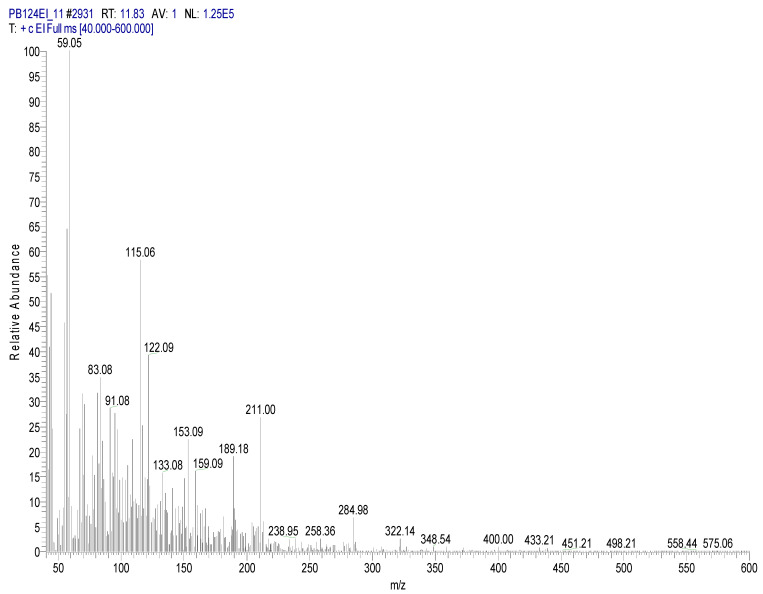
The absence of *o*-chloro benzylidene malononitrile (CBM-C3) at a retention time of 11.83 min, from the sample PB124EI.

In Figure 9, the absence of the *o*-chloro benzylidene malononitrile (CBM-C3) is observed, with a retention time of 11.83 min, based on the sample PB124EI. When comparing the series PA196EI–PA796EI, there is no evidence of the presence of the chemicals of interest, *o*-chlorobenzylidene malononitrile (CBM-C3), *o*-chlorobenzaldehide (C1) or chemicals *o*-chlorobenzylmalononitrile (C2), and 2-(3-chlorobenzylidene)malononitrile (C4).

In the series PB124EI- PB724EI, involving comparison with the CBM solution from the calibration curve, there is no evidence of the presence of the chemicals of interest, *o*-chloro benzylidene malononitrile (CBM-C3), *o*-chlorobenzaldehide (C1), or chemicals *o*-chlorobenzylmalononitrile (C2) and 2-(3-chlorobenzylidene) malononitrile (C4).

### 3.3. Detection via GC-MS of the Main Metabolites of CBM by Hydrolysis Reaction

The degree of hydrolysis of CBM in water was analyzed based on five samples of 20 mg of CBM each dissolved in 40 mL of tap water and extracted with 10 mL of DCM, at different time periods, as follows: CBMHYT0—reference sample, CBMHYT30 (30 min), CBMHYT60 (60 min), CBMHYT90 (90 min), CBMHYT24 (24 h). The toxin did not completely solubilize CBM in tap water, but it exhibited the same volume of extraction solvent for each sample. The chromatographic peak at 2.52 min corresponds to malononitrile (C_3_H_2_N_2_, M 66 g/mol, CAS 109-77-3) and at the retention time of 6.10 min we found *o*-chloro benzaldehide (C1), as hydrolysis products.

The chromatographic peak areas of interest and degree of hydrolysis of CBM in tap water are shown in Table 2.

The content of *o*-chloro benzaldehide (C1) increased from the reference sample, CBMHYT0, from 0.005 mg to 0.402 mg (according to the calibration curve of *o*-chloro benzaldehide (C1) in the sample extracted after 90 min following the contact of CBM with tap water, and 3.59 mg in the sample extracted after 24 h following the contact of CBM with tap water (sample code CBMHYT24h). The obtained results were used to study CBM hydrolysis in the samples that came into contact with the biological suspension.

A comparison was made between the hydrolysis of CBM in tap water, at room temperature (24 h), sample code CBMHYT24h and the sample with biological suspension and CBM, with a period of incubation of 24 h, sample code PB724EI (with a concentration of CBM of 140 µg/mL).

**Figure 10:**
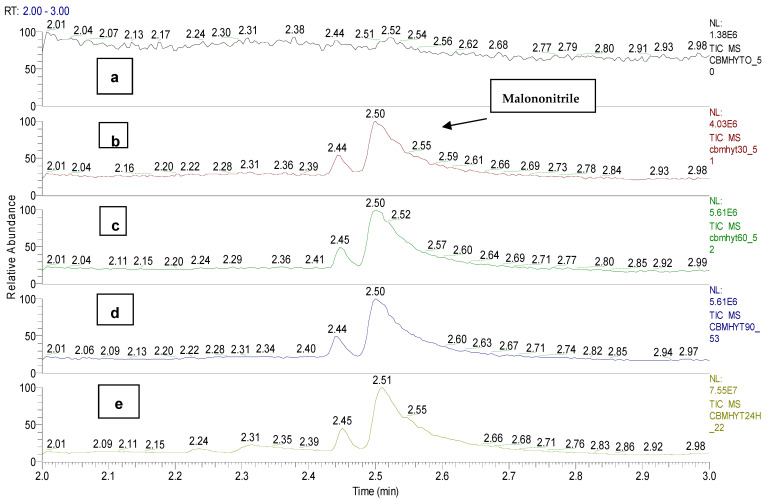
Total ion chromatograms of the organic extracts, coded: (**a**) CBMHYT0, (**b**) CBMHYT30, (**c**) CBMHYT60, (**d**) CBMHYT90, and (**e**) CBMHYT24h domain of 2–3 min; chemical malononitrile with retention time of 2.50 min.

Malononitrile chemical was detected and identified at the retention time of 2.50 min in the samples CBMHYT30, CBMHYT60, CBMHYT90 and CBMHYT24h and the total ion chromatograms are identified in Figure 10.

**Figure 11:**
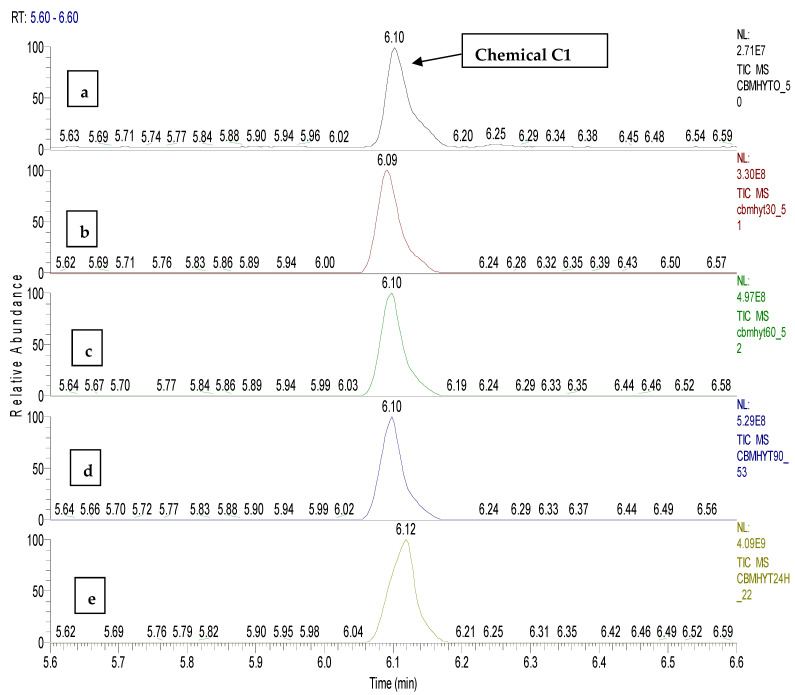
Total ion chromatograms of the organic extracts, coded: (**a**) CBMHYT0, (**b**) CBMHYT30, (**c**) CBMHYT60, (**d**) CBMHYT90, and (**e**) CBMHYT24h, domain of 5.6–6.6 min; chemical *o*-chloro benzaldehide (C1) with retention time of 6.10 min.

*o*-chloro benzaldehide (C1) chemical was detected and identified at the retention time of 6.10 min in samples CBMHYT0, CBMHYT30, CBMHYT60, CBMHYT90 and CBMHYT24h, and the total ion chromatograms are identified in Figure 11.

**Figure 12:**
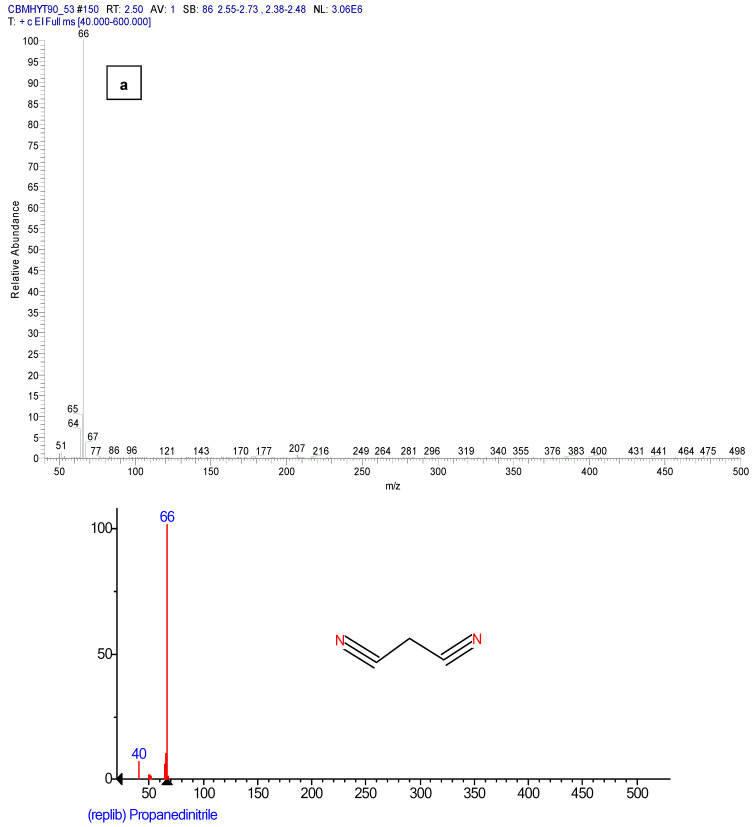

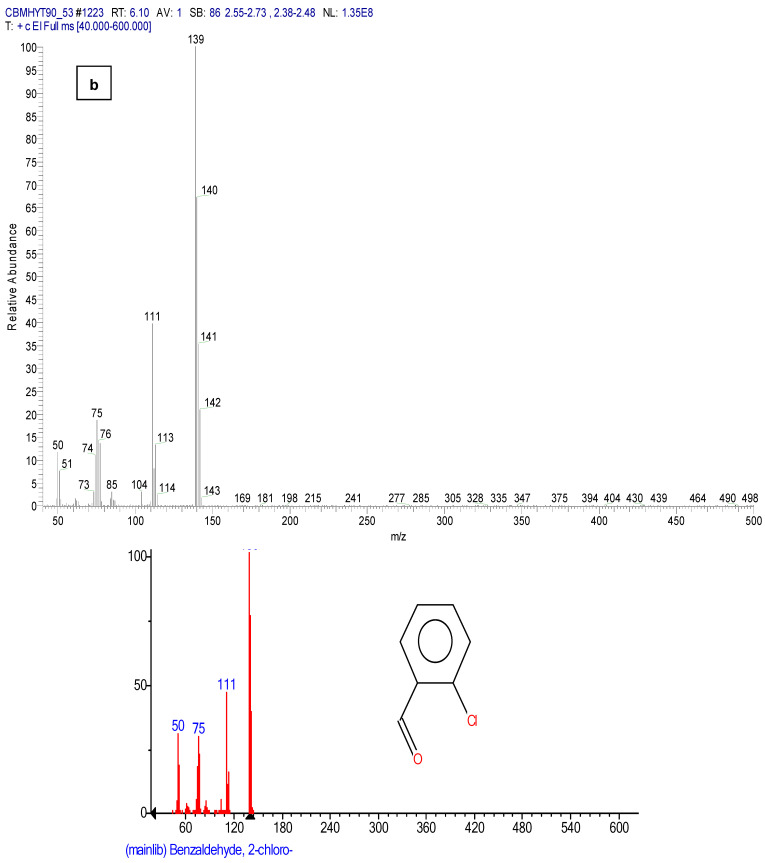
Mass spectra of: (**a**) malononitrile and (**b**) *o*-chloro benzaldehide (C1), from the sample code CBMHYT90, confirmed by the reference chemicals from the NIST database.

Figure 12 shows the mass spectra of malononitrile and *o*-chloro benzaldehide (C1), the hydrolysis products of CBM, confirmed by the reference chemicals from the NIST database.

### 3.4. Comparison between the Hydrolisis of CBM in Tap Water and in the Presence of a Biological Material, Culture of Chlorella sp., at 24 h Period of Contact Time

**Figure 13:**
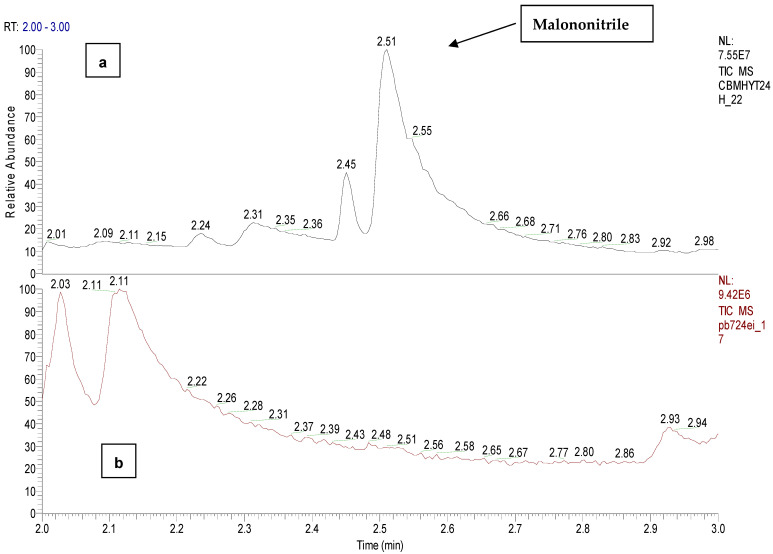
EI total ion chromatogram of the: (**a**) organic extract of a hydrolysis reaction of CBM (24 h), sample code CBMHYT24h and (**b**) the solution of 24 h incubation, sample code PB724EI, domain of 2–3 min. No evidence of malononitrile in PB724EI.

**Figure 14:**
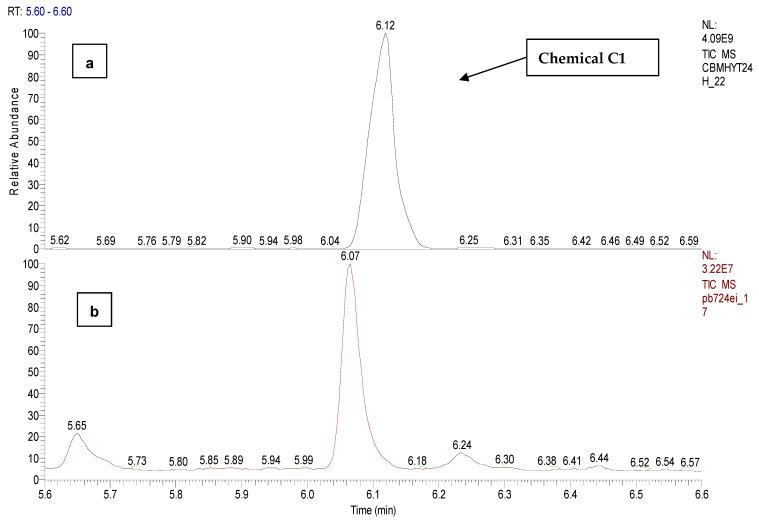
EI total ion chromatogram of the: (**a**) organic extract of a hydrolysis reaction of CBM (24 h) sample code CBMHYT24h and (**b**) the solution of 24 h incubation, sample code PB724EI, domain of 5.6–6.6 min. No evidence of *o*-chlorobenzaldehide (C1) in PB724EI.

Comparison between the hydrolysis of CBM in tap water, room temperature (24 h), sample code CBMHYT24h and the sample with biological suspension and CBM, with a period of incubation of 24 h; the sample code PB724EI is presented in Figure 13 and Figure 14, which compare the hydrolysis of CBM in tap water, room temperature (24 h), the sample code CBMHYT24h and the sample with biological suspension and CBM, with a period of incubation of 24 h, and the sample code PB724EI (with a concentration of CBM of 140 µg/mL).

**Figure 15:**
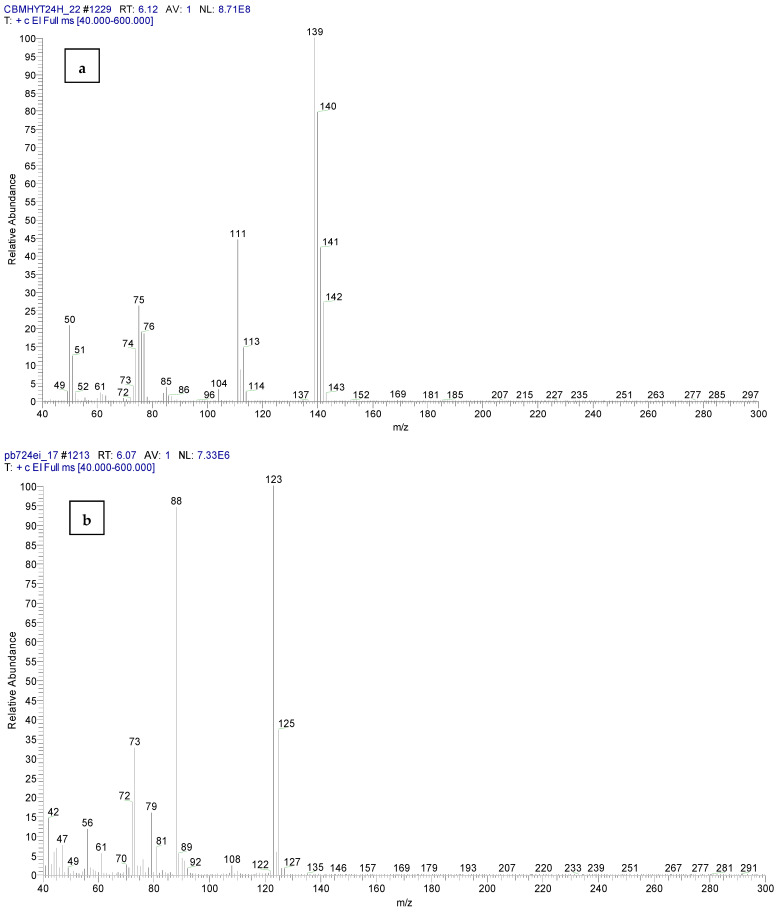
Mass spectra of: (**a**) *o*-chlorobenzaldehide (C1) from CBMHYT24h and (**b**) no evidence of chemical C1 in the sample PB724EI.

Mass spectra of *o*-chlorobenzaldehide (C1) from CBMHYT24h (retention time of 6.12 min) and no evidence of the chemical in PB724EI are presented in Figure 15.

## 4. Discussion

Modeling the behavior of CBM in the environment consists of establishing some mathematical relationships correlating the results obtained based on the way it interacts with the components of the biotic environment. Knowing the behavior of chemicals in the environment is important for assessing the risks that chemicals can generate in ecosystems. Carrying out these experiments allowed the study of the sensitivity of Chlorella algae to different toxic concentrations [45,46].

To carry out these studies, under safe conditions and special security, the laboratory synthesized the CBM compound, whose purity was determined by GC-MS. The chemical composition demonstrated the presence of *o*-chlorobenzaldehyde (C1), *o*-chlor benzyl malononitrile (C2), *o*-chlorobenzylidene malononitrile (CBM-C3) and 2-(3-chloro benzylidene) malononitriles (C4), which were also utilized during the two types of experiments. CBM was used both for spiking water samples that contained the biological material, as well as for carrying out, for comparative purposes, the hydrolysis studies. The domain of concentrations in which it was used, to spike the biological suspensions as well as the hydrolysis studies, was between 20 and 140 µg/mL, respectively, and 500 µg/mL, and the points of the calibration curve were in the range 2–2000 µg/mL.

The separation of the compounds was performed via GC-MS, and the identification and confirmation of the compounds was conducted with the help of the NIST database, based on the explicit presentation of the mass spectra for each compound. The same procedure was followed when preparing the samples containing the biological material as well as the aqueous solutions containing CBM, with the extraction being carried out in both cases with DCM: there was no difference in the extraction time, type of stirring, organic solvent drying and filtration, for the purpose of analysis via GC-MS.

The quantitative analyses were performed in accordance with the calibration curves of both CBM and of *o*-chloro benzaldehyde (C1), and were reported to the reference solutions. In the case of the biodegradation capacity study using microalgae, the reference was the solution containing only the biological material (sample code P10 blank). In the case of the study of the hydrolysis reaction with sampling at different time intervals, the reference solution was the one from time t_0_ (direct contact between CBM and water).

The removal yield of the organic substances present in the water depends on the environmental conditions, the chemical composition of the samples and the chemical substance dissolved in the water that constitutes the substrate for the metabolic activities of the microalgae that use these substances in the biochemical reactions of cellular enzyme complexes. The tests performed indicate that the suspension of *Chlorella* microalgae consumed the entire amount of CBM and metabolites from the analyzed samples. The tests prove that the biological material can be used for the decontamination of the affected areas.

The extraction and analysis of samples containing CBM and biological material, sample code PA196EI-PA796EI (96 h) and PB124EI-PB724EI (24 h), with an incubation period of 96 h or 24 h and different concentrations of CBM, revealed the absence, from absolutely all investigated samples, of CBM as well as its two main metabolites, malononitrile and *o*-chlorobenzaldehyde (C1).

In parallel, the extraction and analysis of the samples resulting from the CBM hydrolysis reaction revealed the presence of two metabolites: malononitrile with a retention time of 2.50 min and *o*-chlorobenzaldehyde (C1) with a retention time of 6.10 min. During these CBM hydrolysis tests, an increase in the content of compound C1 was observed, through quantitative GC-MS determinations, from 0.005 mg in the reference sample (sample code CBMHYT0) to 3.59 mg in the sample extracted after 24 h (sample code CBMHYT24h), according to the calibration curve of C1.

The CBM content decreased from 17.62 mg in the reference sample to 13.49 mg in the sample kept in contact for 24 h, resulting in a CBM hydrolysis degree of only 23.41%. The malononitrile compound showed an increase of approximately 3.5% in the sample exposed for 24 h (sample code CBMHYT24h), compared to the sample which maintained contact for 30 min (sample code CBMHY30). The presence of the two metabolites of CBM in the hydrolysis samples is confirmed and supported by the mass spectra of the two compounds, according to the NIST database.

The biological samples were studied, examined and compared in order to identify the functional groups obtained via FTIR scanning of control sample PA10 compared to sample PA7, which contained 140 µg/mL CBM. The differences obtained indicate FTIR spectra distinct bands and were assigned a range of vibrationally active chemical groups: lipids bands at 2930–2850 cm^−1^, protein amide I band mainly (C=O) stretching 1583–1700 cm^−1^, Protein as (-CH_2_) and as (-CH_3_) bending of methyl, Lipid as (CH_2_) bending of methyl 1425–1477 cm^−1^ and pectin (bands at 1610 cm^−1^, 1424 cm^−1^); Carboxylic group of esters (bands 1720–1700 cm^−1^) [29,30].

After the tests, it was observed that the cellular inhibition of the *Chlorella* sp. microalgae was due to the oxidative stress to which the biological suspension was subjected, which generated a change in the composition of the biomass compared to the control sample that was treated under identical conditions but without the toxin. The differences observed via FTIR scanning showed that in the sample that contained the concentration of 140 µg/mL CBM, weaker signals of specific functional groups were identified compared to the control sample that was not under oxidative stress. However, the concentration of 140 µg/mL did not cause damage to the culture of *Chlorella* sp.

## 5. Conclusions

The results obtained after analyzing the degree of hydrolysis in the samples that contained the biological suspension indicated that no CBM metabolites were detected in any biological sample, regardless of the test concentration, analyzed 24 h after incubation.

CBM in contact with microalgae are used in the biochemical processes in which they are involved. The retention time and biodegradation capacity led to an increased effect of the metabolism of toxic substances. In metabolic reactions, organic substances are the source of carbon and energy for the biochemical processes. Thus, we conclude that the suspension of *Chlorella* sp. consumed the entire amount of CBM from the samples. The tests prove that the biological material can be used to decontaminate the affected areas.

In future tests, we propose to analyze the effect on *Chlorella* sp. of the toxic CBM by studying the minimum inhibitory concentration it generates on axenic cultures, looking for information that could be useful in the medical field for the treatment of specific diseases.

The evaluation of the chemical risk induced by the persistence of chemical substances used in military operations requires the development of economically feasible methods of remediation of contaminated areas. The integration of effective depollution methods that decontaminate and, at the same time, reduce the risks of chemical pollution requires the use of biological methods as a viable solution.

## Figures and Tables

**Table 1:** The sample components, sample code CBM04, detected and identified via GC-MS.

Chemical Name	Retention Time (Minutes)	Molecular Formula	Chemical Structure	Molecular Weight (g/mole)	CAS Registry Number
*o*-chloro benzaldehide (C1)	6.10	C_7_H_5_ClO	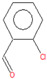	140	89-98-5
*o*-chlorobenzyl malononitrile (C2)	11.31	C_10_H_7_ClN_2_	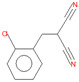	190	40915-55-7
*o*-chloro benzylidene malononitrile (CBM-C3)	11.93	C_10_H_5_ClN_2_	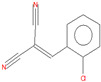	188	2698-41-1
2-(3-chloro benzylidene)malononitrile (C4)	12.82	C_10_H_5_ClN_2_	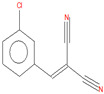	188	2972-73-8

**Table 2:** The hydrolysis grade of CBM in tap water.

Sample Code/Chemical	Malononitrile (tr 2.52 min.)	C1 (tr 6.10 min.)	CBM-C3 (tr 12.14 min.)	Mass of CBM (mg)	Hydrolysis Grade (%) *
	GC area				
CBMHYT0	0	64,347,004	52,890,605,168	17.62	-
CBMHYT30	8,974,787	814,678,251	52,777,364,000	17.58	0.21
CBMHYT60	16,137,050	1,185,660,077	50,024,457,883	16.66	5.42
CBMHYT90	17,355,009	1,257,452,113	49,620,527,787	16.53	6.19
CBMHYT24h	264,863,119	10,837,442,146	40,513,601,453	13.49	23.41

* Hydrolysis grade (%) = (initial mass − final mass)/initial mass × 100/ is calculated according to the calibration curve of CBM.

## Data Availability

Not applicable.
